# Adaptive temporal processing of odor stimuli

**DOI:** 10.1007/s00441-020-03400-9

**Published:** 2021-01-06

**Authors:** Sofia C. Brandão, Marion Silies, Carlotta Martelli

**Affiliations:** Institute of Developmental Biology and Neurobiology, Johannes Gutenberg University, Mainz, Germany

**Keywords:** Olfactory system, Stimulus dynamics, Sensory adaptation

## Abstract

The olfactory system translates chemical signals into neuronal signals that inform behavioral decisions of the animal. Odors are cues for source identity, but if monitored long enough, they can also be used to localize the source. Odor representations should therefore be robust to changing conditions and flexible in order to drive an appropriate behavior. In this review, we aim at discussing the main computations that allow robust and flexible encoding of odor information in the olfactory neural pathway.

## Introduction

Detecting changes in the world is the main challenge for any sensory system. Signals in the form of light, pressure, temperature, or chemicals are rarely static entities for an observer. Either because sensory signals are dynamic, such as cars moving on the street or odors carried by wind, or because of the observer’s movement, there is no stimulus that can be described as static. The external world acquires meaning by connecting causes and effects across different timescales. As a consequence, insight on the function of a sensory system relies on the understanding of how it processes changing stimuli (temporal processing) and how it changes the way it processes stimuli based on their history (adaptation or stimulus-driven plasticity). In this review, we aim at discussing the current understanding of temporal processing and stimulus-driven plasticity in insect olfaction. We focus mostly on peripheral encoding and provide an outlook on how odor stimuli are processed in higher brain regions. We will mostly discuss the *Drosophila* olfactory system, as this is where we have the best mechanistic and functional insight, but also compare with other animal models when relevant, to properly understand brain function.

The olfactory system aids two main behavioral tasks: odor source recognition and localization. Odors rarely exist in isolation and the ability to localize an odor source requires the ability to recognize it across a wide range of stimulus conditions. Recent work in model systems like *Drosophila* and mice has begun to quantify in detail the behavioral responses to odors in different conditions (Álvarez-Salvado et al. 2018; Demir et al. 2020; Radvansky and Dombeck 2018; Tadres and Louis^2020^). Understanding which decision the animal takes in response to a specific stimulus and context is fundamental to infer the underlying neural computations. This leads to the investigation of the neural circuits and molecular mechanisms that implement or support these computations.

**Fig. 1:**
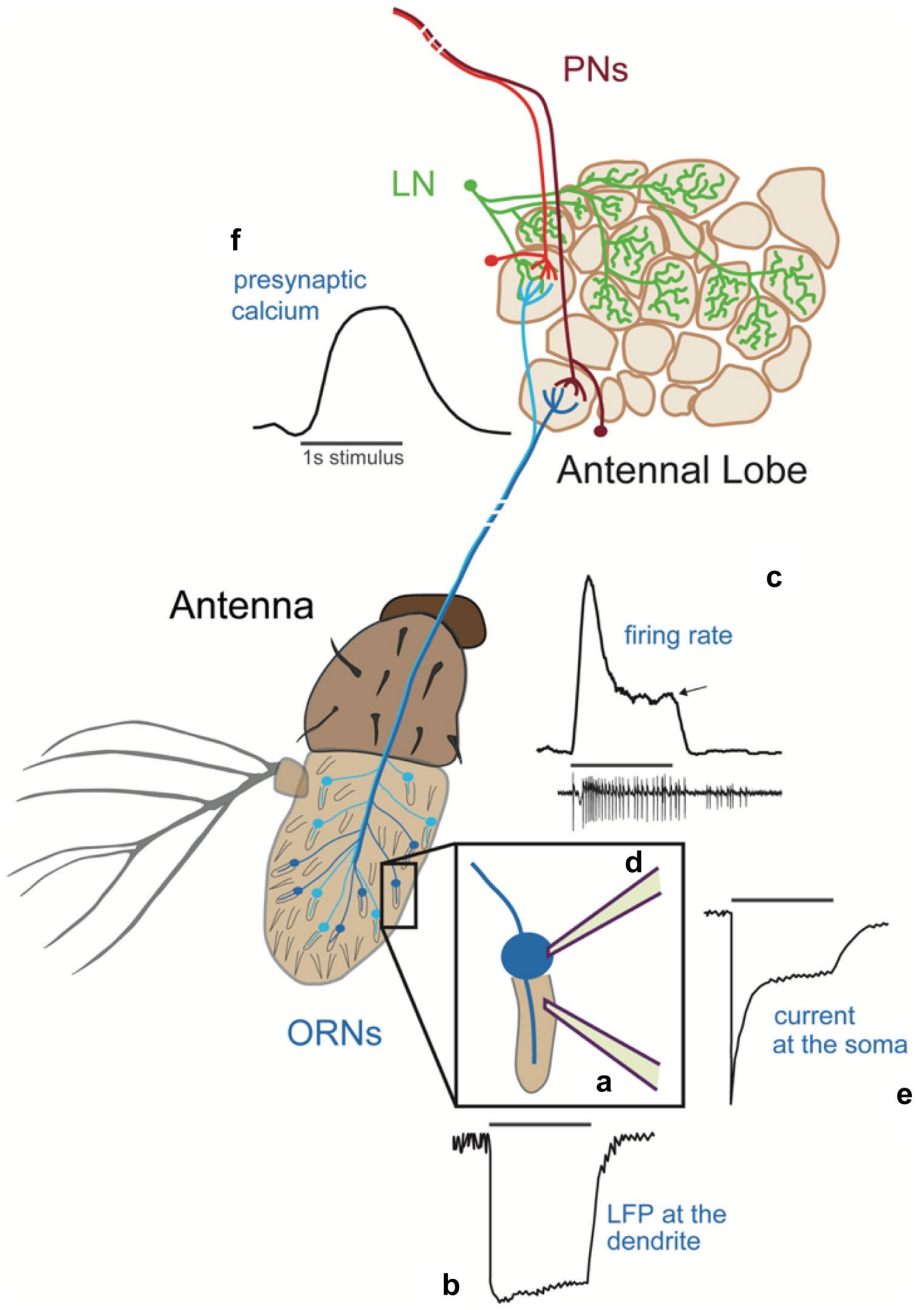
Temporal aspects of the odor response within a single neuron. ORNs (blue) extend their dendrites in hair-like structures called sensilla. Single sensillum recordings (**a**) are carried out in vivo from the intact antenna and allow the quantification of the LFP (**b**) and the spiking activity of the neuron (**c**) (Clyne et al. 1997). In a sliced antenna preparation, it is possible to access the cell body for patch clamp (**d**) (Cao et al. 2016). This technique allows the quantification of somatic and dendritic currents (up to a certain distance from the recording site) (**e**). ORNs expressing the same OR send their axons to a single glomerulus in the antennal lobe, where they make synaptic connections with local neurons (LNs, green) and uniglomerular projection neurons (PNs, red). 2-photon calcium imaging allows the quantification of the presynaptic activity (**f**) reported by the calcium indicator GCaMP genetically expressed in specific ORNs (Riemensperger et al. 2012). All the curves are schematic representations of the neuron response to a 1s odor puff reported by the specified technique. See main text for more detail

The main neural circuit in the insect olfactory system is the antennal lobe (AL), the equivalent of the vertebrate’s olfactory bulb (OB) (Wilson and Mainen 2006). The principles underlying the architecture of the AL and OB are very similar. They both have a glomerular structure resulting from the axonal projections of olfactory receptor neurons (ORNs) and the dendritic arborizations of projection neurons (PNs) (Fig. 1). In the fly, the ORNs are located on two main peripheral organs, the antennae and maxillary palps (Joseph and Carlson 2015). Each ORN type expresses a single or few chemosensory receptors and sends its axon to a single glomerulus of the AL (Fig. 1) (Couto et al. 2005; Fishilevich and Vosshall 2005; Marin et al. 2002). A large class of PNs is uniglomerular and takes input from a single ORN type (Stocker et al. 1990). The glomeruli are therefore parallel functional units whose chemical sensitivity depends on the receptor gene expressed in the ORNs (Hallem and Carlson 2006). These functional units are coupled through a diverse family of local neurons (LNs), most of which are inhibitory (Chou et al. 2010; Liou et al. 2018). Therefore, odor stimuli are first encoded in the activity of single ORNs and this peripheral representation is then integrated and processed centrally in the AL.

What kind of computations should the olfactory system support in order to aid odor source localization? As we will see in the first section, odor concentration changes in time and space, depending on the environmental conditions. The statistics of the stimulus set constraints on which behavioral strategy and neural computation will work best under different conditions. For example, the problem of localizing the source of an odor that diffuses, forming a smooth gradient, has a simple optimal solution: moving up to the highest concentration gradient (chemotaxis) (Gaudry et al. 2012). Therefore, the odor gradient should be computed within the sensory pathway. Does the olfactory system perform such computation? Is this computation implemented in peripheral sensory neurons? Or downstream in the AL?

Sensory systems adapt to stimulus features that remain constant over time, such as mean intensity or variance. The function of adaptation is not to simply decrease responsiveness to a stimulus feature, but rather to maintain a robust sensory representation in different environmental conditions. In vision, for example, adaptation aids contrast invariant response in different luminance conditions (Clark and Demb 2016; Ketkar et al. 2020; Laughlin 1989). Similarly, adaptation in olfaction could support the contrast sensitivity necessary for chemotaxis. But, to what extent we can stretch the analogy between different sensory modalities depends on differences in the type of stimuli and the organization of the corresponding neural circuitries. Both in vision and in olfaction, stimuli are encoded by parallel functional units of receptor neurons or their postsynaptic circuitry. A visual scene is spatially mapped into a retinotopic representation, in which neighboring cells in the visual system encode information from neighboring points in visual space. Correspondingly, in olfaction, a smell is mapped into the combinatorial activation of the ORNs, with similar odors activating similar combinations (Haddad et al. 2008; Malnic et al. 1999). However, while the retina is composed of computational units, photoreceptors, that are identical in terms of the stimuli they detect (number of photons of a given wavelength), ORNs sense stimuli that are chemically different. Downstream of the sensory periphery, the identity of an odor must be reconstructed by pooling information across all the ORNs. So, when it comes to the integration of these distributed inputs, mechanisms for encoding information about changing stimuli could largely diverge in olfaction and vision. In olfaction, adaptive changes in the stimulus representation should retain chemical specificity at least up to the AL, which holds a representation of odor identity. Understanding how adaptation occurs across parallel functional units is therefore key to identify stimulus features that are robustly transmitted to the brain (i.e., intensity, identity, contrast, etc.).

The olfactory system has another peculiarity: the AL constitutes the only sensory processing layer before odor information is integrated with other sensory inputs in higher brain areas, the mushroom body (MB) and the lateral horn (LH) in insects (Jefferis et al. 2007; Lin et al. 2007; Stocker et al. 1997). These brain areas, although serving different functions, must process olfactory information in a stimulus-dependent manner to associate it to other sensory inputs. In order to support context and experience dependent behavioral responses to olfactory stimuli, these brain areas must retain an adaptive and flexible representation of the stimulus.

In this review, we will discuss which neural computations might aid odor source localization by keeping this task robust, chemically specific, and flexible. First, we will talk about odor stimulus properties and their relevance for behavior. Second, we will describe peripheral computations focusing on what stimulus features activate the ORNs and how the ORNs adapt to static characteristics of the stimulus. Third, we will highlight the major computations that occur at the first olfactory processing center. Finally, we will discuss the form of temporal processing and stimulus driven plasticity in higher brain areas involved in multisensory integration.

## The dynamics of odor stimuli and what matters for behavior

Describing the stimuli present in the environment is the first step for understanding which information is available to the animal and inferring what a sensory system is designed to detect. In vision, a quantitative understanding of the statistics of natural scenes has allowed major insight in the function of the visual pathway (Dyakova and Nordström 2017). A quantitative description of the stimulus is certainly what makes the study of the olfactory system unsettling when compared with other sensory modalities. Identifying the specific molecules and relative abundance present in an environment can be accomplished via the analysis of the chemical content of a gas sample. However, obtaining temporal and spatial resolution of a chemical stimulus is an even more complicated issue (for a recent review, see Pannunzi and Nowotny 2019). All mass-based approaches, from the molecular specific GS-MS (gas-chromatography mass-spectroscopy) to the more widely used fast PID (photoionization detector), rely on sample collection and analysis. Therefore, these methods perturb the stimulus, such that the better we sample, the farther from the real stimulus we get. However, these approaches are still useful in laboratory conditions where the chemical composition of a smell can be simplified down to a single component and samples are available in nearly unlimited amounts.

Importantly, not only the absolute concentration, but also the time-dependency of the concentration is odor specific (Andersson et al. 2012; Martelli et al. 2013). Odor-dependent differences in stimulus dynamics can be attributed to two independent processes: liquid source evaporation and surface interaction (Gorur-Shandilya et al. 2019; Pannunzi and Nowotny 2019). These odor-specific properties then couple with the fluid dynamics of the odor delivery system. Even when odors are delivered in a continuous air stream in controlled lab conditions, the output odor concentration will depend on the fluid dynamics of the specific delivery method—which can attenuate or enhance odor-specific differences in the stimulus (Gorur-Shandilya et al. 2019). As a result of these observations, odor stimuli must be measured for every experiment and experimental setup in order to draw conclusions about temporal processing in olfaction. Different dynamics in the stimulus cause measurable differences in the temporal response of the ORNs (Martelli et al. 2013; Su et al. 2011), which necessarily provide additional odor-specific information to higher brain regions. For example, it has been shown that flies can discriminate odors sensed by a single ORN type, likely using temporal information (DasGupta and Waddell 2008). In this study, flies had to discriminate pairs of odors with very different volatility, a parameter that correlates well with the dynamics of the odor stimuli (Martelli et al. 2013). This suggests that temporal differences used for odor learning and discrimination might be already intrinsic to the stimuli themselves, rather than arising from physiological processes at the periphery. Mixtures of inhibitory and excitatory stimuli with different dynamics can elicit mixture-specific temporal patterns of ORN activation (Su et al. 2011), suggesting a possible role for odor-dependent dynamics in the perception of mixtures. The behavioral consequences of odor-specific dynamics in lab assays and in natural conditions remain a relatively new area of research that requires further investigation.

When it comes to quantifying the odor stimulus in more realistic and behaviorally-relevant conditions (e.g., in 2D and 3D assays), measuring odor stimuli by sampling in space and time is not the best solution, unless the stimulus is well reproducible (Álvarez-Salvado et al. 2018). Away from the source and from surfaces, odor stimuli can be modelled as scalar quantities (concentrations) superimposed on a vector field (air speed). Therefore, a possible approach is to neglect odor-specific effects (by using mono-molecular high volatility inert compounds) and to use optical methods for quantifying the spatiotemporal distribution of tracer molecules, such as acetone (Connor et al. 2018) or smoke (Demir et al. 2020). These approaches allow direct spatiotemporal quantification of the stimulus used for behavioral analysis. But what did we learn about the stimuli themselves? As shown by earlier field studies (Cardé and Willis 2008; Murlis and Jones 1981; Murlis et al. 2000; Riffell et al. 2008) and predicted by physical models (Celani et al. 2014), odor signals in turbulent flow conditions are intermittent due to chaotic changes in the speed and direction of wind. This means that the odor concentration measured at a given position downstream of a fixed source will fluctuate abruptly as plumes come by. The statistics of the plumes depend on air speed and geometrical constraints dictated by the environment. Importantly, only those stimuli that exceed the sensitivity threshold of the olfactory system are relevant to behavior. This imposes a biological cut-off on the stimulus statistics, which is equivalently hard to estimate, given the variety of chemicals and the repertoire of sensors available in each animal. In general, average odor concentration decreases and plume intermittency increases with distance from the source (Celani et al. 2014). These different conditions likely induce different levels of adaptation in the olfactory pathway. Although we do not know the true statistics of specific natural stimuli, we expect both a certain degree of flexibility in how odor information is used and a robust encoding of stimulus features at different distances from the source.

When animals localize an odor source, they must deal with and take advantage of whatever information is available. Pure odor-driven behavior is only achieved in the absence of wind and visual cues and, therefore, in conditions where the odor distribution is solely determined by diffusion. In this case, the animal must climb a rather smooth concentration gradient, similarly to what bacteria experience during chemotaxis (Sourjik and Wingreen 2012). In most cases, however, odors do not only diffuse passively, but are also transported by the wind. Wind adds two factors into the problem of odor source localization: (1) it modifies the statistics of odor concentration, which is no longer smoothly changing, but rather flickering, and (2) it engages a second sensory modality: mechano-sensation. In close-to-laminar conditions, when fluid particles move with little mixing, the odor concentration is stable over time and wind direction, detected by the mechano-sensory system, provides a reliable cue for source localization. The detection of an odor will thus induce a surge upwind (Álvarez-Salvado et al. 2018). In flight, surging behavior is also aided by the visual system (Van Breugel and Dickinson 2014). In between the two scenarios of pure chemotaxis vs pure odor-driven upwind surging, a nearly infinite range of conditions exists, for which localization of an odor source can involve a variety of strategies that rely on the integration of olfaction, mechano-sensation and vision (Baker et al. 2018). It could be a general approach of the animal to make choices that maximize the gain of information about the source location (Vergassola et al. 2007). Surely, there is no odor source localization without the olfactory system, but it remains unclear whether and in which range of conditions wind detection is necessary in addition to being useful. Turning upwind at an odor encounter is not an optimal strategy if the turbulence is too high and wind direction uninformative (Demir et al. 2020). Similarly, it is not optimal to try to estimate the mean stimulus intensity when the rate of plume encounter is low (Boie et al. 2018; Victor et al. 2019). Instead, behavioral decisions require a trade-off between odor sampling accuracy and response speed.

But what information about the stimulus is encoded in the olfactory system? We will start from discussing which stimulus features are encoded in the response of ORNs.

## The dynamics of olfactory receptor neuron response

The first step of odor sensing occurs when the odor molecules bind to a chemosensory receptor. ORNs in flies express one or few members of one of five families of chemical sensors (Joseph and Carlson 2015). Here, we focus on ORNs that express members of the odorant receptor (OR) family (Clyne et al. 1999; Gao and Chess 1999; Vosshall et al. 1999). ORs respond to fruit and plants odors, which constitute cues for food sources (Hallem and Carlson 2006). In insects, odors activate a heterodimer of the OR and the OR co-receptor (orco), a receptor complex that functions as an ion channel (Sato et al. 2008; Wicher et al. 2008). Most ORNs express a single OR gene, which confers odor sensitivity and selectivity (Hallem et al. 2004). Odor binding opens the channel inducing membrane depolarization and the ensuing generation of action potentials. ORN responses in *Drosophila* have been quantified using single sensillum recordings (SSR) and patch clamp recordings.

In SSR, a sharp pipette is placed into the sensillum at the level of the dendrite and used to record ORN activity in vivo*,* extracellularly from the intact neuron (Clyne et al. 1997)(Fig. 1a). Most sensilla in *Drosophila* house only two ORNs, which express specific receptors in stereotyped combinations and fire action potential of different shapes. In addition to firing rates, SSR recordings allow quantifying the sensillum local field potential (LFP) and, using receptor-specific ligands, it is possible to attribute the LFP to a single neuron (Nagel and Wilson 2011). The LFP should be representative of transduction events that drive membrane depolarization at the dendrite (Vermeulen and Rospars 2001). As initially reported in moths (Kaissling et al. 1986), the LFP has more tonic dynamics and adapts less than the firing rate (Fig. 1b, c). Qualitatively, similar firing dynamics have been observed in cockroaches (Lemon and Getz 1997) and locusts (Raman et al. 2010), but have been studied in great detail in *Drosophila*. The fly’s ORN responds to a short stimulus pulse (0 → *C*, from zero odor to concentration *C*) with a transient increase in firing rate, which then decreases to an adapted value (Fig. 1c, arrow). At odor offset (*C* → 0), the firing rate transiently reaches zero and then recovers to spontaneous activity. There is enough similarity in ON and OFF firing dynamics that linear-nonlinear models can be used to fit ORN firing response (Martelli et al. 2013; Nagel and Wilson 2011). The ORN response can be well predicted by convoluting the stimulus with a biphasic linear filter coupled to a rectifying non-linearity (Martelli et al. 2013; Nagel and Wilson 2011). The filters extracted from the response to bimodal flickering stimuli (where at each time point the concentration is either 0 or *C*) are similar to those obtained with fluctuating stimuli with Gaussian distributed amplitudes around a mean concentration *C* (Gorur-Shandilya et al. 2017). The presence of a negative lobe in these filters indicates that the ORN can compute temporal differences in concentration. The linear filter has a width of about 200 ms (with small variations due to stimulus statistics), suggesting that this is the typical timescale on which an ORN computes changes in the stimulus. However, this estimate could be an upper bound due to the intrinsic dynamics of the stimulus used. Importantly, ORNs do not calculate a perfect derivative, as the positive and negative lobes of the filter do not sum up to zero. Consistently, on short timescales, the ORN firing rate does not adapt back to basal activity after stimulus onset. ORN firing rate rather adapts proportionally to peak response and with the same degree of adaptation across concentrations and adapted states (Martelli et al. 2013). This is true in spite of transduction kinetics being slower than in non-adapted conditions (Nagel and Wilson 2011). The slower transduction is indeed compensated by faster spike generation (Gorur-Shandilya et al. 2017). When the effect of stimulus kinetics is deconvolved from the ORN response, similar dynamics are observed for different odorants and receptor combinations (Gorur-Shandilya et al. 2017; Martelli et al. 2013). Analogous conclusions were drawn from measurements of calcium responses in the dendrites of larval ORNs (Si et al. 2019). Here, ORN dynamics have slower timescales than those measured in SSR, not only due to the kinetics of the calcium reporter, but also because dendritic calcium might contain a slower component involved in adaptation (discussed below).

There are few exceptions to these rules. First, when stimuli reach saturating concentrations, dynamics can fail to follow the stimulus ON and OFF (Martelli, unpublished). Moreover, there are reports of *Drosophila* (Montague et al. 2011) and mosquito (Turner et al. 2011) ORNs with sustained responses to specific odorants delivered at non-saturating concentrations. A short puff of these odorants elicits minute-long activation of specific ORNs, impairing responses to subsequent stimuli. This phenomenon is possibly due to slow unbinding kinetics of the odor molecule or slow deactivation of the receptor and can have important behavioral consequences by masking response to other stimuli.

Similar to observation in moths (Kaissling et al. 1986), the dynamics of the sensillum LFP in flies is much less transient than the firing rate and can be fitted with monophasic linear filters (Gorur-Shandilya et al. 2017; Nagel and Wilson 2011). This difference between the LFP and firing rate suggested that ORN response dynamics could, at least partially, be determined by mechanisms involved in action potential generation. Knockdown of the Na^+^ channel α-subunit DmNa_v_ makes ORN dynamics more transient (Nagel and Wilson 2011), suggesting that the ratio of Na + /K + conductance is a determinant of the kinetics.

In contrast to the tonic LFP signals, receptor currents measured in patch clamp recordings in antennal slices show transient and strongly adapting kinetics in response to an odor puff (Cao et al. 2016)(Fig. 1d, e). These transient receptor currents depend on calcium influx. In absence of extracellular calcium, the peak current amplitude is also increased, arguing in favor of an immediate feedback of calcium on channel inactivation. We do not know whether the receptor complex and/or other channels are being inactivated, but it seems clear that an adaptive process is visible in the receptor current and not in LFP measurements. Identifying which mechanisms determine ORN dynamics and adaption is important because specific mechanisms impose different computational constraints for stimulus encoding. However, a direct comparison between LFP and current is not straightforward. First of all, the preparation (in vivo intact antenna vs sliced antenna) and the odor delivery methods (gas vs liquid phase) are very different. Second, the LFP is measured at the dendrite, while currents are measured patching the soma at the base of the sensillum; therefore, it is possible that these techniques capture different electrical events. Importantly, different ionic gradients might be present at the dendrites, which bathe in the sensillum lymph, and at the soma, which is surrounded by auxiliary cells (Vermeulen and Rospars 2001). It will be important to compare firing rate dynamics obtained with these techniques to understand how the measured transduction dynamics depend on the stimulus and the physiological conditions or the recording site.

Contrary to what was found for ORNs expressing ORs, ORNs that express ionotropic receptors (IRs) show sustained and non-adaptive currents (Cao et al. 2016). IRs are a more ancient family of chemosensory channels that in *Drosophila* are expressed in dedicated sensilla (coeloconic) (Rytz et al. 2013). Functionally, they are more narrowly tuned and less sensitive than ORs (Silbering et al. 2011; Yao et al. 2005). In SSR, IR-dependent firing rate dynamics adapt less than ORs to consecutive odor pulses (Getahun et al. 2012). However, in contrast to the tonic transduction current, ORNs expressing IRs show relatively phasic spiking responses (Benton et al. 2009) and ectopic expression of IRs in an ORN that normally hosts ORs is sufficient to recapitulate the firing patterns measured in the endogenous neuron (Abuin et al. 2011). This suggests that the cellular context in which these different receptors are expressed is similar and determines to some degree the conversion of the transduction current into firing events. A more detailed comparison of the firing dynamics of IR- and OR-expressing neurons will be useful to understand to what degree their response kinetics and adaptation properties differ and what the relevant steps are that shape firing rate dynamics.

The fast and transient response of ORNs facilitates the quick detection of changes in stimulus concentration. The timing of odor detection has been shown to be very precise across ORNs of the same type distributed on the antenna (Egea-Weiss et al. 2018). Moreover, the speed of odor processing is similarly fast across insect species (Szyszka et al. 2014), suggesting a fundamental function in behavior, for example when insects navigate complex odor plumes. The timing of an odor response is important not only for the quick reaction to a stimulus, but also for stimulus identity encoding. Indeed, ORNs respond with different delays to different odors (Martelli et al. 2013) and concentrations (Egea-Weiss et al. 2018) and this information can be used for the quick recognition of an odor cue (Szyszka et al. 2011). A “primacy coding” scheme has been proposed in vertebrates as a mechanism to encode odor identity in the temporally precise activation of the most sensitive ORNs (Wilson et al. 2017). This mechanism supports a representation of odor that is concentration invariant, since the sequence of activated ORNs is the same across odor concentrations.

## ORN adaptation and its functional consequences

Unicellular organisms like bacteria perform chemotaxis by comparing odor concentrations in space. To do so, they move around and calculate the temporal difference of the ligand concentration sampled at subsequent positions and bias their locomotion towards where the concentration increases (Sourjik and Wingreen 2012). This strategy also works well for more complex organisms in windless environments, with the advantage that they can rely on multiple sensors such as two antennae or two nostrils (Duistermars et al. 2009; Louis et al. 2008; Rajan et al. 2006) and they can move their head for active sampling (Gomez-Marin et al. 2011, Alex et al. 2010). Bilateral comparison clearly requires precise temporal computations downstream of the sensors, but here we will focus on computations downstream of a single pathway.

**Fig. 2:**
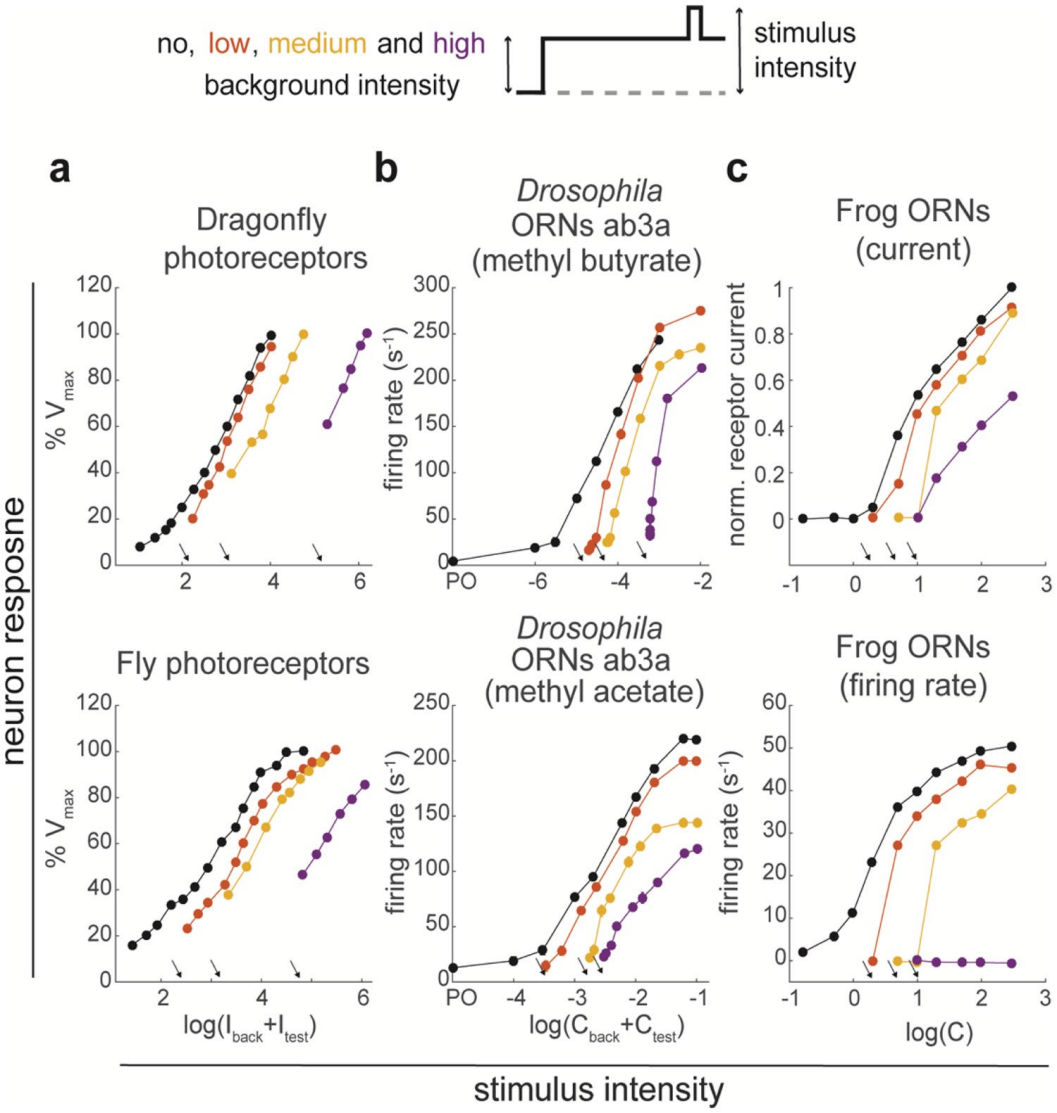
Comparing the effect of background stimulus intensity on the response of light- and odor-sensitive neurons. (**a**) Response of photoreceptors to light stimuli of different intensity in dark-adapted conditions (black) spans three-log units of light intensity. In the presence of an adapting background light (intensity indicated by the arrows), photoreceptor sensitivity shifts up to more than three-log units (from black to purple). Adaptation to the background stimulus increases the basal potential (first data point of each curve) and pushes response saturation to higher light intensities, keeping the response dynamic range centered around the mean stimulus. Data are from Laughlin and Hardie (1978) reproduced with permission of S. Laughlin. (**b**) Response of *Drosophila* ORN ab3A to two odorants at different concentrations. Odor puffs are presented isolated (black) or on a background of the same odorant at concentrations indicated by the arrow. Data are same as in Fig. 4 of Martelli et al. (2013), but plotted as a function of the total stimulus concentration (*C*_back_ + *C*_test_) for comparison with the data of the photoreceptors. Adaptation to the background only slightly increases the basal firing rate (first data point of each curve). The response reaches saturation at lower firing rates and at the same concentration as in no-background conditions. (**c**) Response of frog ORNs: receptor current (top) and firing rate (bottom) from (Reisert and Matthews 1999), reproduced with permission of J. Reisert. Adaptation to the background decrease receptor current and firing rate. The ORN dynamic range does not simply shift to the right, and, for the highest background, the neuron stops firing spikes

Flies can perform chemotaxis with a single ORN type (Louis et al. 2008), suggesting that differences in concentration can be calculated along a single sensory pathway. Calculating changes in the input is one of the basic computations a single neuron or a single synapse can perform (Brunel et al. 2014). To achieve responses across a wide range of stimuli and under different adapted states, changes are ideally calculated relative to the intensity of the background stimulus, the definition of stimulus contrast (Δ*C*/*C*_0_). Sensitivity to relative changes in the stimulus is the main computation that mediates bacterial chemotaxis (Sourjik and Berg 2002). For neurons that respond to stimuli on a logarithmic scale, for example following a Hill function, being contrast sensitive corresponds to shifting the response function to match the background intensity. This shift in logarithmic scale implies a decrease in response gain inversely proportional to the background (Weber-Fechner law). Neurons in the visual pathway can shift their response around the mean luminance such that they can maintain sensitivity to variation in light intensity. For example, insect photoreceptors respond to light intensities covering around three orders of magnitude (Fig. 2a, black dots from (Laughlin and Hardie 1978)). Adaptation to the background shifts this response range along the stimulus intensity axis for at least three-log units (Fig. 2a, colored dots).

A shift in sensitivity is often assumed and postulated as a fundamental computation necessary for odor navigation (Kadakia and Emonet 2019; Victor et al. 2019). Indeed, also ORNs exhibit a different response function when adapted to different background odor intensities (Fig. 2b). However, the decrease in ORN activity cannot be strictly interpreted as a shift. There are three important differences when comparing odor adaptation to light adaptation. First, when adapted to a background concentration, the saturated firing rate is significantly reduced as compared with non-adapted conditions, indicating a decreased coding capacity. Second, ORNs saturate at the same concentrations as in non-adapted conditions. Third, the response function becomes steeper, as also shown in moths (Kaissling et al. 1986). Thus, while photoreceptors effectively shift their dynamic range to match the background stimulation, ORNs decrease their response without shifting it, ending up with a reduced coding capacity. Another property of the ORN response in adapted conditions is the asymmetric coding of increases and decreases in concentration (Δ*C* vs − Δ*C*). We mentioned that on short time-scales ORNs adapt their firing rate proportionally to peak response. However, when the stimulus is presented for longer times (> 10 s), ORN firing rate further decreases to a level closer to baseline activity (Fig. 2b, arrows). As a consequence, in background-adapted conditions, even a small decrease in odor concentration can easily drive the firing rate to zero. Therefore, any decrease in concentration is encoded as a zero, while an increase is encoded depending on its amplitude. In conclusion, in adapted conditions, ORNs respond to changes in concentration, but do not strictly encode stimulus contrast (Cafaro 2016; Kim et al. 2011), and respond asymmetrically to ON and OFF stimuli.

For a comparison, we show the results of similar experiments obtained in earlier studies in frog ORNs (Reisert and Matthews, 1999). Background adaptation reduces both currents and firing rate in response to an odor stimulus, without shifting the concentration at which the neuron reaches saturation (Fig. 2c). Notably, no spikes are fired in response to stimuli presented on a background of intensity within the dynamic range of the blank-adapted ORN. These data have been interpreted as a decrease in ORN sensitivity, because effectively, the ORN cannot respond to low concentrations anymore. It also certainly does not gain coding capacity at higher stimulus intensities, as would happen with a shift of the response function.

So far, we considered pulse-shaped stimuli delivered on a clean air stream or superimposed on a fixed background concentration, conceptually similar to flashes of light in dark or light adapted conditions. However, the neuron response function might be different when tested with different stimulus statistics. Gorur-Shandilya et al. have shown that in response to small fluctuations around a background stimulus, ORN gain decreases proportionally to the inverse of the adapting stimulus, consistent with the Weber-Fechner law (Gorur-Shandilya et al. 2017). The result means that there is a precise relationship between the background concentration and the change that is necessary to elicit an equally-sized response. This holds true for small fluctuation, about sixfold change in amplitude, as reported by PID measurements. But as we have seen, the result cannot be extended to large and quick changes in the stimulus, as those generated by odor pulses superimposed on a fixed background (Fig. 2). This is most likely because large variations drive the neurons response in a more nonlinear regime and out of the mean-adapted condition.

These nonlinearities are partially due to properties of the receptor kinetics itself, as proposed by models (Gorur-Shandilya et al. 2017). Nonetheless, it is conceivable that the ORN response adapts to other statistical features than the mean. One study reported that ORNs adapt their response gain to the variance of a fluctuating stimulus with Gaussian amplitude distribution (Gorur-Shandilya et al. 2017), although another study did not observe any variance adaptation (Kim et al. 2011). An important point to keep in mind is that it is basically impossible to deliver white noise stimuli in olfaction, as changing the concentration requires a finite amount of time that depends also on Δ*C*. Therefore, Gaussian stimuli with the same mean and variance can vary substantially in correlation time. In cockroaches, ORNs respond with different gain to sinusoidal stimuli of different frequency (same mean and amplitude) (Burgstaller and Tichy 2012) due to the interplay between stimulus and adaptation timescales. Moreover, in moths, the same linear-nonlinear model for the ORN response cannot equally well predict the response to temporally structured stimuli with different statistics (Jacob et al. 2017) and differences in the distribution of blanks and whiffs lead to distinct response functions in ORNs (Levakova et al. 2018). These results suggest that different mechanisms are engaged in the response to stimuli with different correlation timescales. More systematic investigation of how stimulus autocorrelation affects ORN gain control could bring insights on the multiple mechanisms that shape ORN adaptation in *Drosophila*.

If we map these observations back to their behavioral relevance, it seems that in the presence of smooth gradients with no or small fluctuations, ORNs can readily encode relative changes in concentration (both ON and OFF) which can be used downstream of the antenna to support chemotaxis. However, in more turbulent conditions, the nature of odor plumes will result in large fluctuations in concentration (0 → *C* → 0 or *C*_0_ → *C*_1_ → *C*_0_). In this case, mean and variance adaptation likely have no time to occur or develop less than in the presence of Gaussian stimuli or possibly on different timescales. Unless saturated, adapted ORNs can still respond fast to increases and decreases of concentration. However, even a long exposure to a stable background does not seem to shift ORN sensitivity, rather the ORN response amplitude and, therefore, its coding capacity are greatly reduced. More insight in what adaptation does or does not do could be brought about by identification of the molecular mechanisms. This will be discussed in the next paragraph.

## Candidate mechanisms of ORN adaptation

We know surprisingly little about the molecular mechanisms that determine ORN response dynamics and adaptation. Although the phasic part of the firing rate response could be linked to mechanisms of spike generation (Nagel and Wilson 2011), adaptation to the mean of the stimulus seems to occur already in the LFP and therefore at the transduction site of the ORN response (Gorur-Shandilya et al. 2017). This is consistent with the adaptive current measurements in patch clamp recordings (Cao et al. 2016). On the contrary, the fact that the ORN response saturation occurs at the same concentrations across adapted conditions (Fig. 2b) suggests that adaptation does not directly affect the receptor activation kinetics, although this has been proposed as a likely adaptive step. In addition, the response mediated by a specific OR can be decreased by activation of a second OR ectopically expressed in the same neuron (Nagel and Wilson 2011). This implies that the activity of the receptor itself is not necessary for adaptation. However, it does not exclude a modulation of the receptor or of the coreceptor Orco. More experiments are needed in order to exclude one or the other mechanism.

Patch clamp recordings demonstrated a role of extracellular calcium in shaping the dynamics of the transduction current mediated by OR activation (Cao et al. 2016). The source(s) of the calcium and the target(s) of the calcium feedback remain unknown. Is calcium that is flowing in through the receptors sufficient to drive adaptation or are there other channels involved? Does this feedback loop directly affect receptor activation or an amplification step? Contrary to evidence in *Drosophila*, earlier studies in moths showed that extracellular calcium is not necessary for a response, but that calcium released intracellularly mediates response termination (Pézier et al. 2007). In *Drosophila*, inositol triphosphate (IP_3_) has been proposed to mediate adaptation, possibly by activation of the IP_3_ receptor and the release of calcium from internal stores (Deshpande et al. 2000). Similar conclusions have been reached by imaging calcium transients in the antennal lobe (Murmu et al. 2011), but these candidate mechanisms need to be confirmed by more detailed and controlled experiments.

ORN odor responses in the fly are dependent on the phosphorylation state of Orco, which is in turn determined by ORN activity (Guo et al. 2017). However, this modulation occurs over long timescales (several minutes) and there is no direct evidence that this mechanism may affect the ORN response dynamics described above. Several other modulatory metabotropic pathways are present in ORNs (Wicher 2018), but they have not been directly linked to stimulus-induced adaptation on the timescales or with the functional role described above.

As it remains unclear where adaptation exactly happens, two types of computational models have been proposed to explain ORN adaptive response. Spike frequency adaptation (SFA) is a common mechanism for neuronal adaptation (Benda and Herz 2003). Although the specific currents involved might differ across neurons, the general mechanism is a slow hyperpolarizing conductance that is activated upon spike generation. Computational studies have proposed SFA as the main adaptation mechanism in ORNs (Farkhooi et al. 2013; Nawrot 2012; Rapp and Nawrot 2020). This inhibitory feedback could in principle involve calcium influx and would be able to reproduce the linear scaling between adapted and peak firing rate (Liu and Wang 2001), a prominent feature of the ORN response (Martelli et al. 2013). However, a direct comparison of these models to experimental data has not been attempted. Another set of models has instead considered adaptation of the transduction current, which implies the feedback of a second messenger (presumably calcium) on the receptor activity (Gorur-Shandilya et al. 2017; Kadakia and Emonet 2019; Lazar and Yeh 2020; Nagel and Wilson 2011). These models can reproduce a number of features of the ORN response to dynamic stimuli, but the molecular basis will need to be validated experimentally.

In conclusion, a role for calcium in shaping the dynamics of ORNs is plausible, but further studies are necessary to identify its source and molecular targets. Computational models can help in this process. To better understand what ORN dynamics and adaptation are good for, we should next consider (1) their role in odor coding and (2) the information processing steps downstream of ORNs.

## Temporal processing and adaptation in the first olfactory processing center

**Fig. 3:**
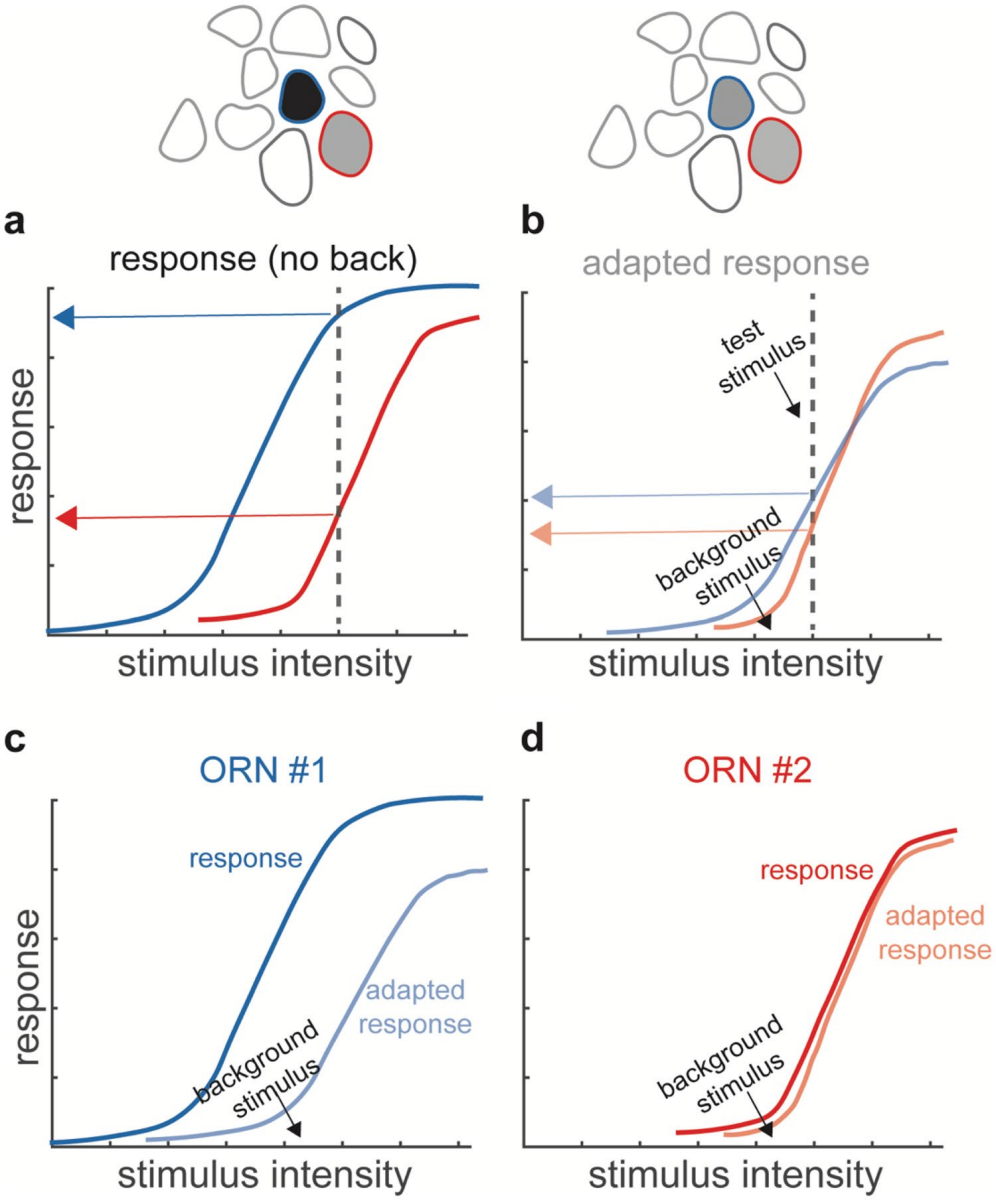
Adaptation changes the combinatorial representation. **a** Response of two ORNs to stimuli of increasing intensities. Red and blue arrows indicate the response of two ORNs to a given stimulus intensity (dotted line). **b** In the presence of a background the odor representation in the two ORNs (red and blue arrows) differs from the non-adapted response (**a**). **c**, **d** A background stimulus, indicated by the black arrow, changes the response function of ORN1. ORN2 is not sensitive to the background stimulus and its response function is unaffected by the background stimulus

The limited capability of single ORNs to adapt their sensitivity is likely not a limitation for the olfactory system. Odors are encoded in the combinatorial activity of a large population of ORNs, each responding with different sensitivity to the specific odor (Hallem et al. 2004). When the odor concentration increases, one ORN might get saturated, but another ORN, expressing an OR with lower affinity to the odor, will be activated. For example, the *Drosophila* larva uses multiple sensors with different sensitivity to the same odor in order to maintain a robust behavioral response over a large range of concentrations (Asahina et al. 2009; Kreher et al. 2008). But how is odor information preserved in the combinatorial activity when the single ORN adapt? And which information is preserved? Consider two ORNs with different sensitivity to the same odorant (Fig. 3a). Here, a background stimulus could elicit adaptation in the more sensitive ORN and therefore a change in its response function, but would leave the response of a less sensitive ORN unaffected (Fig. 3c, d). As a result, peripheral adaptation could change the combinatorial representation of a test stimulus superimposed on the background, possibly confounding the encoding of odor identity (Fig. 3a, b). So, how can we make sense of what an adaptive change in sensitivity implies for the combinatorial code?

This question can be answered by looking at olfactory responses downstream of the antenna, where the axons of ORNs expressing the same receptor form dense synaptic structures called glomeruli (Fig. 1). Within a glomerulus, ORNs connect to PNs and LNs. The network of GABAergic LNs performs a normalization of the incoming ORN odor representation that prevents PN response saturation by adjusting the gain of synaptic transmission ( Olsen and Wilson 2008; Olsen et al. 2010; Root et al. 2008; Silbering and Galizia 2007; Silbering et al. 2008). This transformation is mostly considered an instantaneous computation that involves quick inhibitory feedback from the LNs. However, odor representations in PNs are dynamic and adaptive. In the locust, complex spatiotemporal responses measured in PNs are the result of temporally structured spiking patterns of the ORNs converging on a highly plastic neural network within the AL (Raman et al. 2010). How do dynamic and adaptive ORN representations drive AL output?

Electrophysiological studies in flies have shown that the synapses between ORNs and PNs are strong and depressing (Kazama and Wilson 2008). Short-term depression is a mechanism by which a synapse can compute changes in the presynaptic neuron activity (Abbott et al. 1997). Short-term depression at ORN-PN synapses likely results from vesicle depletion (Kazama and Wilson 2008) and works as a band-pass filter for the transmission of information from ORNs to PNs. Electrophysiological measurements from PNs indeed showed that their response peaks earlier than that of ORNs (Bhandawat et al. 2007) as if they were computing changes in the ORN firing patterns (Kim et al. 2015). Consistent with these observations, PN activity can be fitted by biphasic linear filters (Geffen et al. 2009). Similar conclusions have been reached in studies that compared ORN and PN response to pheromones in moths (Rospars et al. 2014). It remains unclear whether spike frequency adaptation plays an additional role in shaping PN responses, in addition to synaptic depression, as proposed by computational models (Farkhooi et al. 2013).

But how does a phasic firing pattern, like the one from the ORNs, get through such depressing synapses? Differential encoding of the stimulus in the ORN input and synaptic depression at the ORN output constitute two consecutive filtering steps. Computational models show that these two filtering steps would result in very transient PN responses (Nagel et al. 2015). On the contrary, in the fly, prolonged stimulation elicits sustained and stable PN responses. A few additional mechanisms might explain this discrepancy. First, a slow acetylcholine-driven component in the PN response current allows integration of the input over longer timescales making the response more sustained (Nagel et al. 2015). Moreover, transient lateral inhibitory inputs sharpen and stabilize synaptic activity, tuning the effect of incoming firing patterns (Nagel et al. 2015).

To better investigate presynaptic regulation, a recent study combined SSR with imaging of the presynaptic calcium dynamics (Martelli and Fiala 2019). Calcium transients are usually interpreted as a read-out of firing activity. Consistent with this assumption, the amplitude of ORN firing rate measured from the sensillum and the calcium response measured from the presynapses of the same ORN in the AL are strongly correlated in response to short odor pulses delivered in isolation. However, adaptation to the background did not decrease the calcium responses measured at the axon terminals, in spite of an adaptation-dependent decrease in firing rate. Moreover, calcium signals remained sustained (for up to two minutes of odor stimulation), while firing rate was transient (Fig. 1c–f). These non-adaptive dynamics in the presynaptic calcium signal do not seem to rely on inhibitory lateral inputs and point at a role of cell-intrinsic regulation of calcium transients. Additional experiments are needed to understand the mechanistic bases of the calcium regulation and how it is modulated by lateral inputs. From a functional point of view, presynaptic calcium seems to be able to reconstruct the non-adapted ORN response, independently of the strength of the background. This way, the odor representation is preserved at the population level in adapted conditions and does not change over repeated or sustained stimulations (Martelli and Fiala 2019). A direct comparison between firing rate and presynaptic calcium is difficult in vertebrates, because it is hard to reach the olfactory epithelium in a live preparation. However, there are indications of similar differences between peripheral firing adaptation and ORN calcium activity in the olfactory bulb. Indeed, ORN response is suppressed for odor stimuli presented at frequencies comparable with the respiration rate (Ghatpande and Reisert 2011; Zufall and Leinders-Zufall 2000). In contrast, calcium transients in ORN axon terminals measured in vivo report more sustained ORN responses (Carey et al. 2009; Lecoq et al. 2009; Pírez and Wachowiak 2008; Storace and Cohen 2019; Verhagen et al. 2007).

These observations would fit in a scenario where adaptation occurs (or reoccurs) at the level of the antennal lobe, as a coordinated change across all glomeruli. Calcium imaging from *Drosophila* PNs indeed shows that the odor representations at the population level are adapted to the stimulus statistics and encode stimulus contrast, preserving information about the stimulus identity (Martelli and Fiala 2019). The quantification of synaptic release showed that a slow component of the PN response dynamics originates presynaptically, through depletion of vesicle release and independently of the calcium dynamics (Martelli and Fiala 2019). This slow component is consistent with models of vesicle recycling that involve two pools of vesicles (Hallermann et al. 2010).

Adaptive processes through the AL almost certainly involve LNs. LNs are an extremely diverse population of neurons which differ both anatomically and functionally (Chou et al. 2010; Liou et al. 2018; Nagel and Wilson 2016; Seki et al. 2010). They are not very selective to odor identity, but they show different preference to features of the stimulus (ON vs OFF) depending on the input they receive. Moreover, their temporal dynamics are extremely diverse depending on intrinsic cellular properties (Nagel and Wilson 2016). This means that the effective amount of inhibition a glomerulus receives will depend on the type of LN that innervates it in combination with the stimulus dynamics. Inputs from a fast LN will drive strong transient inhibition when the stimulus strength steps up or down, while slow LNs will drive more tonic inhibition through the entire stimulus presentation. Furthermore, a considerable subset of LNs has patchy morphology connecting only subsets of glomeruli (Chou et al. 2010) and not all glomeruli are equally sensitive to inhibition (Hong and Wilson 2015). Additionally, some commonly used LN driver lines seem to be dispensable for an odor response to isolated pulses (Strube-Bloss et al. 2017), indicating that they might have a function in other stimulus conditions. Although adaptation has not been directly studied in LNs, we expect that the rich dynamics and tuning of inhibitory inputs play a role in adapting the PN odor representation to the statistics of the stimulus. Lateral inhibition in the AL mediates a divisive normalization of the input from ORNs, which rescales PN activity based on the overall activation of the AL (Olsen and Wilson 2008; Olsen et al. 2010). What happens to this transformation when stimuli are sustained or repeated remains an open question. Work in moths shows that ORN adaptation drives a shift in frequency of odor-induced oscillations in the AL, suggesting a role of adaptation in engaging lateral inhibition to synchronize the PNs population dynamics (Ito et al. 2009). Moreover, early studies in the locust have shown that plasticity in the AL driven by repeated stimulus presentations decreases PN activity, but increases firing precision and population synchrony (Stopfer and Laurent 1999). This is independent of ORN adaptation, occurs at different time scales, and could be mediated by tuning of the inhibitory synapses (Bazhenov et al. 2005).

AL plasticity also occurs on longer timescales. Hour- and days-long exposure to odors induces reversible changes in the activity of specific glomeruli and reduces behavioral response selectively to these odors (Das et al. 2011; Devaud et al. 2001; Sachse et al. 2007; Sadanandappa et al. 2013; Sudhakaran et al. 2012). These forms of long- and short-term habituation have been attributed to the selective potentiation of recurrent GABAergic inhibition specifically onto PNs (reviewed in (Ramaswami 2014)).

Overall, these studies suggest that different subsets of LNs have different functions and operate on a large range of timescales. Connectomics data will provide useful information to investigate the computational function of LN subsets (Bates et al. 2020; Berck et al. 2016; Horne et al. 2018). Targeted behavioral experiments should finally aim to disentangle the different functions that lateral inhibition seems to mediate adaptive and robust encoding of stimulus features, modulation of odor preference, and context integration.

## Stimulus history modulates odor representations in higher brain regions

Downstream of the AL, PNs form synapses onto the Kenyon cells of the mushroom body (MB) and lateral horn neurons (LHNs). How do odor-driven dynamics and plasticity in PNs affect the representation of odor information in these downstream neurons? LHNs have been shown to be faster and more accurate in responding to odors compared with their presynaptic PNs (Jeanne and Wilson 2015). This is due to a dynamic spike threshold that is activity dependent and enables the detection of simultaneously incoming spikes. It remains to be investigated how these physiological properties adapt to sustained or repeated stimulation. LHNs have been recently characterized in terms of their anatomical properties and odor response spectrum (Frechter et al. 2019; Jeanne et al. 2018). This neuron population is incredibly diverse suggesting that it supports different functions. Current models suggest that the LH mediates innate behavior and olfactory navigation (Schultzhaus et al. 2017). However, further studies should be aimed at understanding which specific LH neurons are involved in navigation and which computations and stimulus features are used in the LH to help the fly localize an odor source.

Stimulus-driven plasticity in the olfactory system might have multiple roles. So far, we have discussed how this plasticity can support robust function by keeping an invariant representation of stimulus features of possible behavioral relevance. However, when looking at neural computations downstream of the AL and their behavioral implications, we must distinguish between what the fly can do and what the fly wants to do. On the one hand, stimulus-driven plasticity could support robust encoding of stimulus features for goal-directed odor tracking. A recent theoretical study showed that adaptation in the olfactory pathway supports sparse odor representations in the MB, facilitating the quick recall of memories necessary to drive animals during navigation (Rapp and Nawrot 2020). On the other hand, stimulus-driven plasticity could support flexible representations that allow the fly to change its goals. In flies, the short-term history of the chemical composition of an odor scene can change the animal behavioral response to an odor (Badel et al. 2016). A model of preference normalization based on olfactory context suggests a plastic role in the circuit downstream of the AL in integrating PN output (Badel et al. 2016). Whether this type of normalization happens in the LH or in the MB is unclear. In their behavioral assay, Badel et al. did not associate odors to a reinforcement or to a behavioral outcome, rather the odor preference seems to rescale based on the valence of the previously experienced chemical context. This phenomenon indicates a form of stimulus-driven short-term plasticity which could in principle involve either the MB or the LH. That stimulus context shapes MB output has been shown in several studies (Bräcker et al. 2013; Lewis et al. 2015), but whether context integration is adaptive and on what timescales past stimuli are integrated remains to be clarified.

Stimulus-driven adaptation is a mechanism that enhances the encoding of novel features of a stimulus. On short timescales, novelty can be an increase or a decrease in concentration, but on longer timescales, novelty can depend on how familiar the animal is with the stimulus. At least one mushroom body output neuron in the fly encodes the novelty of an odor stimulus (Hattori et al. 2017) and requires odor-driven activity in dopaminergic neurons. While sensory representations must be stable to reliably report stimulus information, the brain must remain flexible to what that information means for the animal. Stimulus-driven plasticity in brain areas that support learning has been previously reported in rats (Best and Wilson 2004) and more recently in zebrafish (Jacobson et al. 2018). Changes in the odor representation in higher order areas are sometimes referred to as adaptation, habituation or plasticity. The common aspect is that they are driven by stimulus history, which in turn indicates that the underlying neural substrates must contain information about stimulus history and filter sensory information based on stimulus history. These changes in the coding space could either lead to changes in behavior (flexibility) or support behavioral invariance (robustness). Which of the two scenarios occurs depends on the specific local and downstream circuit.

## Remarks and conclusions

At the sensory periphery, the encoding of the olfactory stimuli consists of two main steps: the activation of individual ORNs in specific combinations and the coordinated integration of this combinatorial input within the AL. Here, we have discussed in detail the temporal aspects of these encoding steps, with a main focus on their adaptive features. However, our understanding of this encoding process is still lacking essential insights.

First of all, it would be beneficial to understand which molecular mechanisms mediate adaptation of the ORN response. Although limited compared with other sensory modalities, the ORN response function changes depending on stimulus history, which means that based on stimulus history, the ORN selects which information should be transmitted downstream. Mechanisms of olfactory adaptation are better understood in vertebrates, but the kind of computation they mediate has been extensively debated (Reisert and Zhao 2011). Therefore, across organisms, a clear function for ORN adaption is still missing.

A missing tile in understanding the role of adaptation in olfaction is certainly the link between molecular mechanisms, neural function and behavioral output. The tools to investigate these links are one strength of model systems like *Drosophila*, but in the specific context of stimulus-driven adaptation, little has been done. Whether a certain neural computation, e.g. stimulus differentiation, plays a functional role in behavior, such as climbing odor gradients, needs to be investigated experimentally. Having more mechanistic insights on adaptation will make it easier to link computation to function.

Similarly, it will be important to study behaviorally relevant stimuli using electrophysiology and imaging, since basic design principles of the olfactory system might be hidden by using simplified stimulation protocols (like single puffs). There is a trade-off between generating naturalistic and reproducible stimuli in the lab, but new approaches are being developed to control and measure the stimuli delivered.

One conclusion we would like to draw here is that in order to understand adaptation in olfaction, one must look downstream of the receptor neurons. The combinatorial aspect of odor coding requires integration of information across parallel channels. This central integration is what makes the olfactory system different from a chemical sensor, like a single cell. The AL network is likely doing more than a global divisive normalization. The diversity of LNs alone speaks for multiple functions, selective integration of incoming stimuli, and temporal processing on different timescales. The mechanisms of stimulus induced plasticity in this network and their role in olfactory navigation deserve further investigation.

Finally, odor perception involves also long-term state-dependent modulation. How the internal state affects peripheral odor representations has been analyzed so far only in terms of response amplitude to isolated odor pulses (Ko et al. 2015; Martelli et al. 2017). Is hunger only shaping the valence of an olfactory perception? Or is a hungry fly better at localizing an odor because of enhanced peripheral processing? Currently, there are no answers to these questions. However, one interesting possibility is that the internal or behavioral state may directly modulate odor processing in the AL, similarly to how the behavioral state shapes visual processing (Maimon 2011).

Current theories of adaptive behavior suggest a role for top-down modulation of sensory perception (Młynarski and Hermundstad 2018). This means that not only behavioral states, like resting or walking, but also behavioral output could potentially affect fundamental sensory computations. After an evaluation of the smell has been made, different strategies might be used to localize an odor source (Baker et al. 2018; Gaudry et al. 2012). Therefore, behavioral output might feedback onto odor processing in order to adjust or improve the search strategy. 

New tools for stimulus quantification and behavioral analysis are being developed and implemented to study how animals use olfaction to move around aimfully. These approaches will allow the field to address long-standing fundamental questions concerning the identification of neural computations that mediate animal decision-making in the presence of odor cues.
